# Lectures on Clinical Medicine, Delivered at the Philadelphia Medical Institute

**Published:** 1838-09-12

**Authors:** W. W. Gerhard

**Affiliations:** Physician to the Philadelphia Hospital, &c.


### CLINICAL LECTURES.

### LECTURES ON CLINICAL MEDICINE, *delivered at the Philadelphia Medical Institute, by* W. W. GERHARD, M. D., *Physician to the Philadelphia Hospital*, &c.

#### CHRONIC LARYNGITIS—TREATMENT.

(Continued from page 273.)

THE treatment of chronic laryngitis is always a matter of great difficulty; indeed, when the case has become complicated with tuberculous disease of the lungs, you will find no plan of treatment of much avail. These cases of consumption are the most intractable, and most painful to the patient. It is of little moment whether the disease of the throat has occurred very early, or whether it is merely one of the sequelæ of phthisis; in neither can you hope to succeed in arresting the disease of the throat, if ulceration has once commenced. You have seen enough of these complaints, to understand that a cure is scarcely possible, when the interior of the larynx is constantly irritated by the passage of diseased secretions from the lungs, and by the motion caused by either speaking or deglutition. Still a cure does sometimes take place spontaneously, and may be aided by a judicious treatment.

I once saw a case of most complete cicatrization of the larynx, where the vocal chords had been completely destroyed, and the epiglottis was contracted much within its usual dimensions. The voice, of course, was not restored, but the patient was quite healthy, and died of a disease in no wise connected with the larynx. If the destruction has been complete, you must not therefore look for entire restoration of the functions of the larynx, but for the cessation of the local pain felt in coughing and swallowing, as the test of the disease having quite abandoned its acute character. With the cessation of the pain, there is an end of the acute form of the disease which is attended with active inflammation; but there is a form of chronic laryngitis in which the uneasiness is so slight as not to be felt by patients of obtuse sensations. In these cases, the proof of entire cicatrization is very difficult of attainment. You can only judge in an approximative manner, by the diminution of the hoarseness of the voice, and by the cessation of the harsh, stridulous, laryngeal cough, as well as the disappearance of the clotted, opaque sputa, which are formed in the larynx.

The cases that you have just seen of phthisis, accompanied with chronic laryngitis, were not of the most favourable kind for treatment. Still, in both cases, there were results of a very positive and gratifying character. One patient left the hospital with the symptoms of phthisis much mitigated, and the laryngeal affection decidedly improved. He will not recover, but the relief which he obtained is in itself a most desirable object. The other patient was still more decidedly benefitted; he has no soreness of the throat, and can speak almost in his natural key. These cases exemplify the only treatment for affections of the larynx which I conceive to be called for; I had almost said, which was justifiable in an advanced stage of phthisis. I directed for both of them merely inhalations of laudanum and water, to be repeated several times a day. About a drachm of laudanum was poured on some boiling water in an open vessel, and the patient was directed to breathe over the cup twice daily. This mode has some advantages in such cases over any inhaler; it is preferable to using any vessel with a spout, as it avoids all straining or forcing of the muscles of respiration, and at the same time the patient receives a sufficient portion of the aqueous vapour charged with alcohol and the narcotic properties of the opium. You may readily renew the heat by placing a spirit lamp occasionally beneath the vessel containing the liquid. The vapour of tar, which has been so much lauded in the treatment of phthisis, is often of great service in these cases, but is most useful in those varieties in which the inflammation extends over the pharynx, and thence into the larynx and trachea. I am not disposed to exaggerate the value of inhalations,—they have been greatly abused, or at least have been extolled much beyond their value; but they certainly furnish the most direct mode of acting upon the lining membrane of the air passages, and may be managed so as to avoid all risk to the patient.

In these cases, leeches are often advisable; but they should be applied rarely and in small quantities,—for a large abstraction of blood by leeches applied over the trachea, debilitates extremely, and should be avoided at an advanced stage of phthisis. Now the very reverse of this obtains in the commencement of the very same disease; but two or three days since, I prescribed leeches above the summit of the sternum for a young lady who labours under the commencement of phthisis; the relief from a most distressing sensation of tickling and itching throughout the windpipe, was immediate. I have seen similar results very generally follow in such cases, and regard the application of leeches as much more certainly useful in laryngitis and inflammation of the trachea, than in most cases of disease; but you must always carefully select the proper time and mode of application, or you will find it positively hurtful. I would give you the following practical rule: when there is soreness of the larynx and trachea, and pain on pressure, without the permanent alteration of the voice and appearance of the sputa indicative of ulceration, you may take from two to six ounces of blood, with almost certain advantage. The soreness of the larynx which accompanies the clergyman's sore throat, is nearly as certainly relieved as that which is confined to the peculiar affections of this organ. This leeching may be repeated, if you find that the patient is not rapidly losing his colour, or showing other signs of excessive loss of blood. You must not, however, expect that when extensive ulceration of the mucous membrane of the larynx has taken place, and still less when the cartilages are necrosed, that the abstraction of the blood from the neighbouring parts, will cure the disease; it can no more change this state of things, than arrest the progress of white swelling when the cartilages of the knee-joint are partially eroded. There is nothing to be done, but to check as far as possible the irritation of the part by appropriate inhalations, and then trust to the gradual operation of nature in throwing off the diseased portion. In the syphilitic variety, a mercurial course may be most useful; indeed, you may sometimes succeed in curing cases of the most unpromising appearance, provided they depend upon a venereal cause.

In the late work of Trousseau and Belloc, on chronic laryngitis, which obtained a prize from the Academy of Medicine, much stress is laid upon the cauterization of the larynx. This may seem a bold procedure, and doubtless requires much skill to avoid mischievous effects; but if the operation be practised with care, these authors state that it is not very difficult, and is of great service. They sometimes touch the mucous membrane of the larynx, at its entrance, with the nitrate of silver, but more frequently sprinkle the interior of this organ with a solution of the caustic, by means of a syringe constructed for the purpose. Of course, the great difficulty in the operation consists in introducing the injection behind the epiglottis, and in preventing the caustic from disorganizing the membrane of the larynx, or irritating the adjacent bronchial tubes. I have never attempted to perform this operation, and cannot therefore speak to you from personal knowledge of its powers; but if I were to form any opinion upon the subject, I should conclude from the remarkably beneficial effects of inhalations, that it was a useful mode of treatment in the hands of those who are skilful enough to employ it. It has one great advantage; it irritates but little the bronchial tubes.

External irritants are recommended, perhaps in part from a kind of habit, which makes them always enter into the list of remedies advised for all chronic inflammations. That they are often grossly abused, no one can doubt; and we may regret that we have no means of distinguishing with certainty those cases which are really benefitted by external applications, from those in which they are either useless or injurious. The subject, however, is perfectly open for observation, and may be readily settled on a rigidly determined basis by careful observation. My own experience enables me to give you the following rules as tolerably well fixed. Blisters are rarely useful; I usually avoid them, and would restrict their use to the sub-acute forms of laryngitis; I have seen little benefit, and often much injury, result from their use in the very acute and in the chronic cases. Caustic issues and setons are both troublesome and painful, and rarely of benefit. Frictions over the larynx and trachea, with a stimulating liniment, particularly one so mild as to allow of gentle and long-continued application, are much more useful; indeed, you will often find them of signal advantage in cases of sub-acute laryngitis, and sometimes of service in the more intractable chronic varieties. Weak sinapisms, frequently applied, are also useful, and less inconvenient than liniments.

There are many less important medicinal agents which I am in the habit of using: some of them you have seen me prescribe. These are chiefly the opiates and demulcents; although these medicines are regarded as mere palliatives, they are nevertheless highly important, and prevent the increase of the laryngeal affection. Nothing acts more injuriously upon the larynx, or is of more immediate injury to it, than frequent coughing,—and any simple remedy which can check the constant disposition to cough, is something more than a palliative—it prevents that constant motion of the larynx which hastens the progress of incurable cases of laryngitis, and is a serious obstacle to the recovery of those which are less advanced. The opiates are advantageously combined with ipecacuanha. I greatly prefer the form of lozenge; a medicine which dissolves very slowly, acts more certainly and more completely relieves the distressing sensation of tickling about the entrance of the larynx. You may give a lozenge four or five times daily, containing from the twentieth to the twenty-fifth of a grain of sulphate of morphine, with gr. ¼ to gr. ss. of ipecacuanha; to this I sometimes add a minute portion of antimony.

Whatever treatment you adopt in chronic laryngitis, you will soon find that in no disease is it more necessary to attend rigidly to those hygienic rules, which are here often so much overlooked. The larynx, from its structure and position, is extremely exposed to the causes of disease; and when its mucous coat is ulcerated, or its cartilages denuded, no cure can reasonably be anticipated, unless you protect the organ from the deleterious action of external causes, and keep it as nearly as possible in a state of rest. It is therefore a matter of absolute necessity to keep the patient as silent as possible, and to guard him from sudden changes of temperature. A damp, moist air, is often soothing to the larynx, and rather diminishes the tendency to cough; but if the air be damp, and at the same time chilly, as is the case when easterly winds prevail in this climate, patients with laryngitis nearly always suffer, and cough much more frequently than at milder seasons. A very dry, cold air, produces very variable effects; if the patient be extremely feeble, it is usually injurious, and proves directly debilitating; but if his strength be still retained, the influence of cold, dry air, is no otherwise injurious than as a direct irritant to the larynx. Extremes of heat are injurious on other grounds,—a patient debilitated by the intense heat of summer always suffers from an increase of laryngeal symptoms—he is besides liable to profuse perspiration, which is suddenly checked by draughts of air. Now, from all these causes of disease, it will be your duty to protect the patient, more carefully, perhaps, when suffering from laryngitis, than any other disease; the extraordinary susceptibility of the larynx renders it difficult to restore it to the normal condition when seriously affected.

Silence should be enforced in all acute inflammations of the larynx; but in its chronic diseases, absolute silence is impracticable. All that we can do, is to direct the patient to speak as little as practicable, and to avoid all loud or prolonged exertion of the voice. When recovery takes place, you must permit your patient to return but slowly to his usual tone of voice and habits of speaking, otherwise he will be exposed to a continual recurrence of this most troublesome disorder.

---

### BRONCHITIS.

Bronchitis is an affection, which is rare during the summer months of the year. Most of the cases which occur in our wards are met with in the winter season; and they are generally engrafted on some other affection, for we have few instances of pure acute bronchitis in hospital practice. In its simple ordinary form, it is too slight a disease to require much attention, and in the large majority of cases is left to the unaided efforts of nature. Patients affected with phthisis will tell you, that they laboured first under a bronchitis, which may have been ordinary or secondary. They usually suffer it to run on until it reaches a stage which compels them to seek for medical relief, and they then enter the hospital. Hence in hospital practice, we see only severe and grave cases, which, in many points of view, is advantageous in the study of disease.

We have a case now in the hospital, which is rather unusual: it is not perfectly pure bronchitis, for, as I have said, that is comparatively rare, but it is complicated only with slight pleurisy. The patient is extremely stupid, and we were therefore not able to gather from him much of his anterior history. He is a stout and strong man, a labourer, born in Ireland, entered the hospital the 24th of June. He had been in the out-wards of the house from the 15th of April till the 13th of June, when he went out of the house, and went to work at Havre de Grace. He had no cough of severity until he was hurt on the 21st of June by a bank of sand falling on his breast, and principally on the left side, which became immediately painful, and soon after he became oppressed; he was not bled. Two days after he came to Philadelphia on the rail-road cars, without suffering much pain, and entered the hospital on the 23d; he has been subject to colds, but never had one of any severity. On the 24th, the day after his admission, his condition was the following:

Intellect extremely dull, and memory bad; face slightly œdematous under the eye-lids; feet not swollen, voice hoarse, cough loose and hoarse, appetite lost, slight pain in the left side of the breast, chiefly under the axilla; none in the right side; this pain is increased by breathing; dyspnœa caused by breathing and speaking; skin moist and cool; pulse eighty-four, quick and thrilling; respiration twenty; chest anteriorly full; percussion clear throughout anteriorly and posteriorly, but less marked on the left than on the right, and the left side not so full as the right; sonorous and mucous rhonchi on both sides, anteriorly respiration vesicular and slightly feeble; ordered

Mist. pectoral f℥v.

Syrup scillar. f℥i.

M. S. ℥ss. q. h. secundâ.

*June 25th.*—Man much oppressed; face flushed; dorsal decubitus; loose mucous cough. Pain extending over the sternum, especially towards left side; pulse ninety-six, rather full and resisting; tongue moist, a little whitish; cephalalgia in the morning, and at night very severe, preventing sleep. Sonorous rhonchus extending throughout the whole of the chest; mucous rhonchus in the lower third of the left side; percussion at the base of the right side a little less clear than at the left; the pain was in the right side, but the mucous rhonchus at the left. Venesection—tartarized antimony, two grains dissolved in a quart of flaxseed tea, to be taken during the day and night.

From the 25th to the 29th, the oppression and pain in the right side ceased completely; the flush of the face disappeared. The patient was cupped upon the chest after the bleeding.

*June 29th.*—More oppressed; sweating profuse on the 28th. No pain in the right side, which has not returned since the bleeding; cough very loose; soreness at the upper part of the sternum severe in coughing. Tongue moist; appetite bad. Chill last evening. No palpitation. Pulse eighty-four, rather feeble, regular. Perspiration twelve. Impulse of the heart clear; both sounds clear, distinct, but distant. Anteriorly mucous rhonchus over the whole left side of the chest; vesicular on the right where there was no rhonchus; percussion posteriorly clear on both sides, nearly equal, a little in favour of the right; abundant mucous rhonchus at the lower portion of the left side, with sibilant less marked; no stools; no nausea for the last twenty-four hours.

*July 2d.*—Sweating abundant; pulse eighty-eight; still loose mucous cough; expectoration almost ceased; tongue a little dry at the edges only; one stool yesterday; perspiration decidedly acid, but less so than that of a healthy individual. Abundant mucous rhonchus in the posterior part of the whole chest, particularly on the right side extending throughout, anteriorly respiration feeble throughout. On the 3d, still cough, but more loose; less dyspnœa. Sweating abundant; a little hoarseness; pain only at the thorax; urinates with difficulty and pain; costive; perspiration now alkaline; sputa decidedly alkaline; urine extremely acid; appetite lost; tongue moist; pulse sixty-four, full and soft; mucous rhonchus throughout posteriorly; the tartarized antimony continued.

*July 6th.*—Expectoration muco-purulent, not numular; skin cool; pulse regular, moderately frequent; on the left side anteriorly, the respiration is vesicular and pure; sibilant rhonchus at the summit of the right side, with a little rudeness of respiration; percussion clear. Balsam of copaiba gtt. v. four times a day.

*July 7th.*—Bad taste in the mouth; strength feeble; expectoration thick, yellow, and muco-purulent; twenty drops of elixir vitriol four times a day.

*July 9th.*—Respiration vesicular throughout the left side; traces of sibilant rhonchus only throughout the right side anteriorly; moist and dry rhonchi abundant; percussion sonorous; sleep interrupted by the cough; sweating profuse; anorexia; thirst; two stools in twenty-four hours; tongue a deep purple, but rather dry; strength feeble; pulse ninety-two; saliva slightly acid, although patient has not taken the elixir of vitriol for four hours; perspiration also slightly acid; treatment continued.

*July 10th.*—Perspiration and saliva slightly acid; urine extremely acid; took last night by mistake about twenty drops of copaiba; elixir vitriol continued. The oppression continues; anteriorly on the left side respiration clearer; mucous, with some sibilant rhonchus on the right side.

*July 11th.*—Cough frequent; sweating at night; drowsiness throughout the day; expectoration puriform, thick, running together; twenty drops of laudanum at night.

*July 18th.*—Cough at night, less during the day; skin cool; pulse eighty-eight, feeble; tongue red, smooth, and clear; appetite bad; sweating last night; one stool in twenty-four hours; pain under the right clavicle; vesicular respiration; imperfect sonorous rhonchus at the internal margin; vesicular simply inferiorly; on the left side fuller and more vesicular; no constant expiration; impulse of the heart increased; sounds clear, the second a little dull; percussion clear on both sides, less so on the right; posteriorly on the right side abundance of sonorous and sibilant rhonchi, rather feeble on the left. Dry cups no. viii. between the shoulders.

*July 20th.*—The following prescription was ordered:

℞. Copaiba ʒj.

Tr. Opii ʒss.

Syr. Tolu ℥j.

Mucil. Acac. q. s., ut fiant ℥iv., S. ℥ss. q. h. sec. nocte.

This case, which affords us a very good, although not an uncomplicated example of bronchitis, will enable you to learn the signs of the disease, and the difficulties which sometimes arise from its complication with more grave, though less apparent disorders. The patient was quite well until he was injured by a fall of earth; from that time he began to cough, and at his entrance the bronchitis was fully developed. Now, this is by no means the usual mode of commencement for bronchitis; and if the patient had given a less connected account of his case, I should certainly regard his statement as very doubtful. As it is, you may well hesitate before you admit entirely the account which he has given you; for his perceptions are dull, and he is therefore not capable of appreciating the slight degree of uneasiness which would arise from a previous chronic bronchitis. Of one thing, however, we are certain; that is, that the bronchitis assumed a character of much severity only after the occurrence of the accident, which the patient met with while at his work; the same external violence therefore gave rise to an inflammation of a serous and a mucous tissue.

The diagnostic characters of bronchitis are well illustrated by the present case; the rhonchi which are so well marked in the present case, and which vary from one moment to another, are, on the whole, the best signs of the disease. But you must not lay too much stress on these signs; you must remember the anatomical condition of the bronchial membrane, and keep in view the causes of the rhonchi. Now, in bronchitis, especially if the case be of moderate intensity, such as is offered by this patient, the membrane is throughout its whole extent more or less thickened and coated with mucus of considerable tenacity. The thickening arises from congestion of the blood vessels of the membrane, and will be found to vary very much at different times of the day and in different portions of the membrane; hence the sounds will cease entirely, and be replaced by a respiration which is nearly natural, but more feeble than usual, for the mucous membrane is always sufficiently altered to prevent the respiration from returning to its full strength as long as any inflammation whatever remains. Hence I am disposed to lay more stress upon the feebleness of the respiration, particularly the irregular and varying feebleness, than upon the rhonchi. The feebleness of the respiration is of course most evident when complicated with emphysema; in the emphysematous portions of the lung there is a permanent cause for the feeble vesicular murmur, and they yield scarcely any sound, when the patient has an attack of acute bronchitis added to the chronic disease. You will find that persons affected with emphysema, are especially subject to bronchitis. Besides the feebleness of respiration, those of you who are good auscultators, and I am glad to say that several of my pupils deserve to be so called, must have perceived that the respiration has more or less of the character which I denominate rustling; that is, the inspiratory sound has lost, in a measure, its softness, and a rustling sound is heard during the passage of the air into the smaller tubes and vesicles. This is a very frequent sound in chronic bronchitis; it is also heard in the acute variety, if it happen to be accompanied with but little mucous secretion. By the rustling sound I do not mean the dry crepitant rhonchus, which is rarely heard in these cases, and is therefore an unimportant sign,—but I allude to a mere alteration in the tone of the respiration, no adventitious sound being produced.

You are well aware of the changes which occur in the rhonchi; these are in proportion to the quantity of the secretion into the bronchial tubes, and to the degree of the thickening of the membrane. Thus you observed in the present instance that the mucous rhonchus was well marked when the sputa became abundant; now, in cases of acute bronchitis, it is important to attend to this rhonchus, for when secretion has fairly taken place, you may regard your patient as decidedly improved; but in chronic bronchitis, it is of less importance, except in cases where a chronic dry catarrh is replaced by an acute inflammation: the mucous rhonchus then becomes a very good measure of the degree of severity of the acute disease, and from its gradual subsidence we can ascertain the precise progress made by the lungs in returning to their habitual state.

You may have remarked, that the oppression in this patient was much greater than the physical signs would seem to indicate. Now, when you find this state of things in bronchitis, you may look for its cause in one of two complications; that is, emphysema, or disease of the heart. Of course I exclude complications of an acute kind, such as pneumonia, or severe pleurisy, for slight pleurisy, not more severe than that offered by this patient, does not constitute a very distressing complication. The signs of emphysema, I shall detail to you at another period; they are in part obscured by those of the bronchitis, but still a sufficient number of signs will remain for the diagnosis. The evidence of cardiac disease is rather more obscure; that is, of a moderate degree of enlargement of the heart, without either extensive valvular disease, or inflammation of the membranes. The means of diagnosis are in a great degree within your reach, but it requires much tact and some experience. In the present instance you will scarcely find demonstrable signs of heart disease; at least these are limited to a slight degree of dulness at the bronchial region, and a little confusion of the sounds of the heart: by this expression I mean that the sounds of the heart have not their usual clearness and fulness, although they are not positively so different from the ordinary standard as to be called morbid. The impulse is besides too diffused, and not sufficiently sharp; not limited to the point of the heart; it is, however, at least as forcible as in the natural state. Now, these signs, which I merely allude to, at present, without expecting you fully to appreciate their value, indicate a distension of the heart with blood, causing a laboured and slow contraction, and sometimes terminating by the formation of fibrinous coagula in the right ventricle and auricle. In more favourable cases, this distension of the heart is of little immediate danger, but may lay the foundation for future hypertrophy and dilatation.

Having now pointed out the signs which are important for the diagnosis of this case, I have but a few words to say as to its prognosis: it is almost necessarily favourable, for the complications do not threaten any immediate danger; and the patient, from his age and constitution, is nearly exempt from pulmonary phthisis, which is apt to follow chronic catarrh, in the patients who have any predisposition to the formation of tubercles from hereditary or other causes. I shall insist upon the relations between phthisis and bronchitis, and point out their distinctive characters in another part of the course.

The treatment consisted, as you know, chiefly in venesection, cupping, and tartarized antimony, followed by the balsam of copaiba. The bleeding was particularly requisite in this case, from the full, plethoric condition of the patient, and his evident tendency to congestion of the heart and lungs. When the necessity for a remedy is as strong as in the present instance, you may expect to find that immediate relief will follow its employment. Accordingly, the patient was immediately relieved of his most troublesome symptoms, and especially of his extreme dyspnœa. I am not, however, an advocate for bleeding in ordinary cases of bronchitis; indeed, it often retards the cure,—for in all inflammatory diseases, but more especially those of a secreting mucous membrane, a certain degree of energy in the circulation is necessary to bring about the natural termination of the disease. This termination always takes place by secretion from the inflamed surface, unless the inflammation be arrested so easily as to leave no thickening or congestion of the mucous coat. The disadvantage of bleeding is, that it does somewhat retard the process of secretion, if the bronchitis be not severe, or if the strength of the patient be not very robust; on the other hand, when the congestion is so considerable as to impede the circulation, we find it highly expedient to take blood from the arm. It removes the over large quantity of blood from the heart and lungs, allows these organs to perform their functions with comparative facility, and materially assists secretion. You have seen how materially this was promoted in the present instance, and that the patient was immediately relieved after bleeding. Cupping is less useful in bronchitis, than it is in either pneumonia or pleurisy; the relief is not proportionate to the quantity of blood taken. I use cups but rarely in acute bronchitis; I almost limit their use to those cases in which it is complicated with so considerable a degree of dyspnœa, that bleeding has failed to remove it. They are, however, more useful in the chronic variety; they should then always be applied between the shoulders, a point where you may abstract more blood, and at the same time approach much more nearly to the bronchial tubes.

The tartarized antimony was also directed for this patient; it certainly reduced the force of the pulse, and was probably useful. You need not be startled when you hear me use the words, probably useful; I am most anxious to point out to you the best modes of treatment, and to insist most strongly upon the positive results obtained from treatment, either in the management or cure of disease; but when I do not perceive unequivocal benefit follow from a prescription, I feel myself bound not only not to conceal, but to call your attention to it. In the present instance, we can merely say that our patient improved a little while taking the antimony, uncombined with other remedies; but the medicine was not followed by the same quick resolution of the disease, as often occurs in cases of pneumonia, treated by this remedy. Nevertheless, antimony is, in general, one of our best and most certain remedies in the management of acute bronchitis.

Vegetable emetics are largely used in the treatment of bronchitis; they are most useful in the chronic varieties, or in the bronchitis of children. Squill, ipecacuanha, and nauseants of a more stimulating character, are all used. I shall speak of them in their appropriate place.

The patient is now using the balsam of copaiba, a most excellent article in sub-acute bronchitis. Of the various terebinthinate articles, none is so much used as this balsam; and where it is not resisted by the stomach, its action is more certain than that of any remedy of this class. It probably acts upon the same principle which renders stimulants effectual in the declining stage of most inflammations. It certainly is one of the most certain remedies we possess in the treatment of chronic bronchitis, especially those varieties in which the secretion from the bronchial tubes is much diminished. In the chronic mucous catarrh, its action is less certain, but often highly beneficial. Our patient will, in all probability, require no other treatment, as he is already fast approaching towards convalescence. Other modes will be appropriately mentioned, when I speak to you of the chronic forms of the disease.

